# The effect of autopolyploidy on population genetic signals of hard sweeps

**DOI:** 10.1098/rsbl.2019.0796

**Published:** 2020-02-26

**Authors:** Patrick Monnahan, Yaniv Brandvain

**Affiliations:** Microbial and Plant Genetics Institute, University of Minnesota, St Paul, MN 55108, USA

**Keywords:** polyploidy, hitchhiking, linked selection, hard sweep

## Abstract

Searching for population genomic signals left behind by positive selection is a major focus of evolutionary biology, particularly as sequencing technologies develop and costs decline. The effect of the number of chromosome copies (i.e. ploidy) on the manifestation of these signals remains an outstanding question, despite a wide appreciation of ploidy being a fundamental parameter governing numerous biological processes. We clarify the principal forces governing the differential manifestation and persistence of the selection signal by separating the effects of polyploidy on the rates of fixation versus rates of diversity (i.e. mutation and recombination) using coalescent simulations. We explore the major consequences of polyploidy, finding a more localized signal, greater dependence on dominance and longer persistence of the signal following fixation, and discuss what this means for within- and across ploidy inference on the strength and prevalence of selective sweeps. As genomic advances continue to open doors for interrogating natural systems, simulations such as this aid our ability to interpret and compare data across ploidy levels.

## 1. Introduction

Whole genome duplication (i.e. polyploidization) is common, particularly within plants [1]. This macromutation can impact both macroevolutionary processes of colonization, speciation and extinction [2], and microevolutionary processes of mutation, drift and natural selection [3]. Modern sequencing technologies provide insight into how selection shapes the genomic landscape of divergence and could allow researchers to test hypotheses about how polyploidy alters the tempo and mode of adaptation. However, before we can analyse sequence data to test such hypotheses, we must understand how the same selective pressures alter our ability to recognize a sweep in polyploids.

Numerous features of polyploids could impact the tempo and mode of adaptation. For example, all else equal, an increase in chromosome number will increase the mutational target size, and thus, the rate of adaptation in a mutation-limited world [4]. The additional set of chromosomes may also change dominance and epistatic relationships, potentially offering novel routes to adaptation [5]. Finally, these extra chromosomes can shock the genomic system, initiating manifold internal selective pressures, related to proper regulation of gene expression and chromosome pairing during meiosis [6]. With the ongoing development of sequencing and computational tools, researchers increasingly turn to population genomic studies to better understand the evolutionary consequences of polyploidy and to address hypotheses concerning the nature of adaptation in polyploids [7,8].

**Figure 1.**
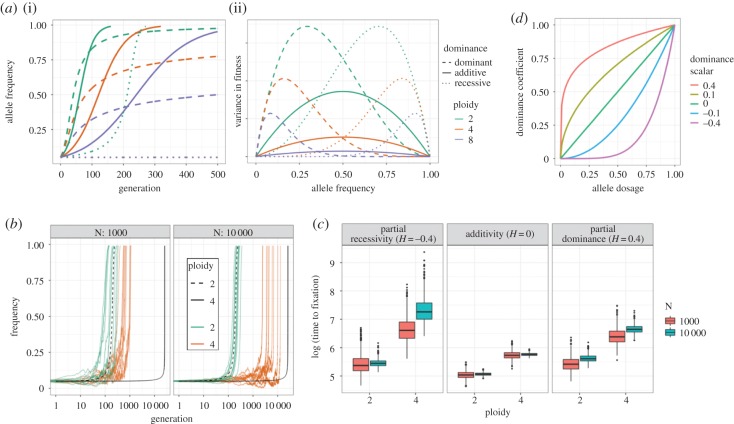
(*a*) (i) Slower rates of allele frequency change (AFC) in higher ploidies. (ii) Reduced variance in fitness in higher ploidies at intermediate allele frequencies. *s* = 0.1. (*b*) AFC for recessive mutations (*H* = −0.4) at different population sizes (*N*). Black lines illustrate the expectation with infinite population size (broken line = diploids; solid = tetraploids). (*c*) Effects of population size on fixation time for different dominance scenarios. (*d*) Dominance coefficients at different allele dosage (expressed as a frequency) for different values of the dominance scalar.

However, scans for selection have been developed with diploids in mind, which hinders the study of selection in natural polyploids. Thus, while we would like to know if polyploidy fundamentally alters selection, we first need to know how two inherent features of polyploidy—larger population mutation and recombination rates (via increased effective chromosome number), and slower responses to selection (due to lower variance in fitness)—change the neutral variation around adaptive substitutions, and consequently, our power to identify and localize sweeps. Importantly, the effect of ploidy on each of these factors is to promote the retention of neutral diversity in polyploids, although they likely differ in their qualitative and quantitative effects. In contrast with the more straightforward, ‘factor-of-two' effects of polyploidy on mutation and recombination, the effect on selection is more complicated due to the added potential for an allele to be masked or diluted in heterozygous genotypes, effectively dampening the single-locus selection response across much of the range of dominance conditions and allele frequency (figure 1*a*) [9].

With appropriate consideration, coalescent simulations can generate haplotype data for an arbitrary ploidy level [10] and thus guide interpretation of patterns of sequence variation. The implicit assumption of free recombination among haplotypes restricts our analysis to autopolyploids (where chromosomal copies derive from a single ancestral species), which, by some estimates are as or more frequent than allopolyploids (resulting from hybridization) [11].

We use these simulations to disentangle how the multifarious effects of chromosome number discussed above impact population genomic signals left behind by selection. We show that the effects of selection on neutral diversity are more striking and locally restricted in polyploids as compared to diploids. Further analyses reveal that the limited reach of selective sweeps in polyploids is more attributable to the extended fixation time, rather than their increased population recombination rates. The differential effects of ploidy on the selection signal are also highly dependent on the dominance of the mutation, particularly for recessive cases. Lastly, we find that the signal of selection persists for more or less time in higher ploidies, depending on the particular metric used. In sum, we highlight the many ways that chromosome number fundamentally alters the selective process and the consequences that this has for population genomic inference and comparison across ploidy levels.

## 2. Methods

### (a) Simulating polymorphism

**Figure 2.**
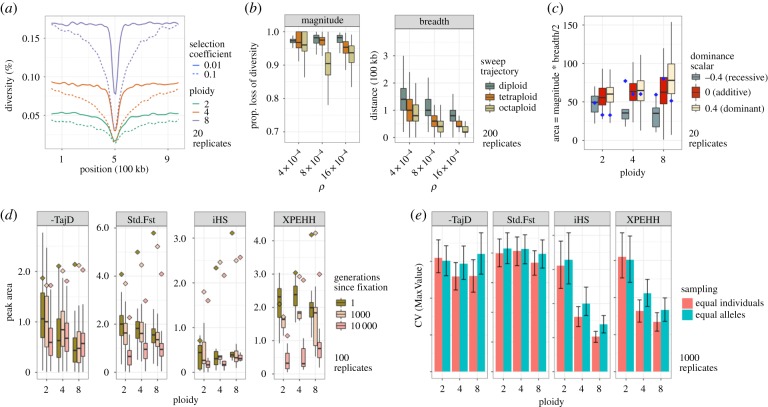
(*a*) Smoothed-profile (LOESS; span = 0.1) of diversity for varying ploidy and selection coefficient. (*b*) Impacts of diversity/recombination and fixation rates on reductions in diversity. (*c*) Ploidy-dependent effects of dominance on diversity (*N* = 10 000). Points denote median for *N* = 1000, multiplied by 10. (*d*) Effect of ploidy on the persistence of selection signals. Points denote median across replicates of maximum values, and boxplots (median, interquartile range (IQR), and 1.5 * IQR) represent the area under the peak. *H* = 0. (*e*) Coefficient of variation for maximum value observed for each replicate. Error bars are from resampling 200 replicates 1000 times. For all plots, *N* = 10 000; *s* = 0.01.

We simulate selective sweeps with *mssel* [12], assuming that polyploidy simply increases the number of chromosome copies, *k* [10,13], and thus the population mutation and recombination rates (*θ* = 2*Nkμ* and *ρ* = 2*Nkr*, respectively). We assume no preferential pairing between homologues, random mating (no self-fertilization/inbreeding) and no ‘double-reduction' (i.e. segregation of sister chromatids into the same gamete) [14]. We further assume populations are at equilibrium prior to selection, remain constant in size and that equal numbers of individuals, *n* (=10, except figure 2*e*), are sampled for all ploidies (i.e. n * k haplotypes; see electronic supplementary material, figure S1 for additional simulation details). We simulate a simple demographic scenario in which an ancestral population splits in two, at which point a beneficial mutation arises in the middle of a 1 Mb sequence (freely recombining, non-centromeric) in one population and ultimately fixes (additional details, below). We sample haplotypes from both populations immediately following fixation (except figure 2*d*), using the non-selected population as a neutral baseline and for calculation of between-population measures of selection (F_ST_ and XP-EHH [15]).

### (b) Generating allele frequency trajectories

Within *mssel,* selection can be specified by an allele frequency trajectory. For a population of arbitrary ploidy (*k*, where diploids = 2, tetraploids = 4, etc.) with allele frequency $p_{t}$, the expected allele frequency in the next generation, ${\hat{\;p}}_{t+1}$, is:

$${\hat{\;p}}_{t+1}=\frac{\sum_{i=0}^{k}\left(\begin{array}{c} k\\ i \end{array}\right)\frac{k-i}{k}p_{t}^{k-i}{\left(1-p_{t}\right)}^{i}(1+h_{i}s)}{\sum_{i=0}^{k}\left(\begin{array}{c} k\\ i \end{array}\right)p_{t}^{k-i}{\left(1-p_{t}\right)}^{i}(1+h_{i}s)},$$

where *i* is number of ancestral alleles, *h_i_* is the dominance coefficient for the genotype with *i* out of *k* ancestral alleles and *s* is the selection coefficient. Mutations arise in single copy, such that initial frequency is 1/kN.

From this expectation, we find allele frequency in the next generation, $p_{t+1},$ as

$$p_{t+1}=1-\;\frac{\sum_{\;j=1}^{N}Binom(k,1-{\hat{\;p}}_{t+1})}{Nk},$$

(Figure 1*b,c*). We iterate this process until fixation or loss, recording only those trajectories leading to fixation.

To compare across ploidies, we assume that the selection coefficient for a beneficial mutation is the difference in relative fitness between alternative homozygotes, that the dominance coefficient for a heterozygous genotype is a simple function of the frequency of beneficial alleles within the individual, and this function is constant across ploidy levels. We use a single value, termed the *dominance scalar* (*H*), to control the form of the dominance function (figure 1*d*; similar to [4]). Given *H* (−0.5 < *H* < 0.5)*,* a genotype's dominance coefficient is $h_{i}={\left(i/k\right)}^{\tau }$, where $\tau =|{\left(10H\right)}^{\mathrm{sgn}(H)}|$. With additivity (*H* = 0), the relationship is linear, whereas the negative (recessive) values of *H* (i.e. *τ* > 1) produce a convex function, and positive (dominant) values result in concavity (i.e. 0 < *τ* < 1).

### (c) Analysis

The numerous metrics designed to detect selection vary widely in assumptions and robustness to confounding forces (e.g. demography). To make valid comparisons across ploidy, we are restricted to metrics not assuming diploidy. We chose two common metrics based on nucleotide diversity (Tajima's D and F_ST_) and two haplotype-based metrics: iHS [16] and XPEHH [15]. We calculated pairwise nucleotide diversity (*π*), F_ST_, and Tajima's D in overlapping windows (step size of 1/2 full window size) using the R package *PopGenome* [17]. After experimenting, we found 1 Mb/(*N/*50) windows, where *N* is the population size, captured sufficient polymorphism for robust calculation of summary statistics across all parameters investigated. To ease comparison among metrics, we mean-standardize F_ST_ (difference from mean, divided by standard deviation) within each replicate to set null expectation to 0, as for other metrics, and also multiply Tajima's D by −1 (so all metrics are on positive scale). We calculated iHS and XPEHH with the R package, *rehh* [18], using the *parse_ms()* function from msr (https://github.com/vsbuffalo/msr) for file format conversion.

Modern genome scans routinely emphasize outlier values along with a visual profile of various metrics along a chromosome as evidence of selective sweeps. This motivates our focus on maximum observed values (i.e. magnitude) and/or the area under the peak when describing our results. For diversity, ‘Magnitude' is calculated as the difference between diversity at bottom of dip and baseline levels in a non-selected population, ‘Breadth' as the distance where diversity recovers to ½ baseline levels (divided by 100 kb) and ‘Area' as ‘Magnitude' * ‘Breadth'/2. For remaining metrics, we calculate area under the peak (±100 kb from selected site; *auc*() function, *R* package *MESS*; scaled by 100 000 for ease of visualization) as well as the maximum values. Code for all simulation, analysis, and visualizations is available at https://github.com/pmonnahan/PloidySim. See [19] for data accessibility.

## 3. Results and discussion

### (a) Effect of ploidy on patterns of diversity following a selective sweep

While higher ploidies exhibit larger absolute reductions in diversity (due to higher baseline diversity levels; figure 2*a*; see electronic supplementary material, figure S2 for individual replicates), it is more locally restricted, whereas the signal is spread over a longer physical region in diploids (figure 2*b*). Assuming the adaptive substitution rate does not differ across ploidy levels, a greater proportion of the genome will thus be affected by selective sweeps at lower ploidies. However, both the elevated recombination rates and time to fixation in higher ploidies (represented by ‘Sweep Trajectory') had a pronounced effect on sweep breadth and the proportionate loss of diversity (figure 2*b*). Together, this suggests that, with whole genome sequencing, sweeps will be better localized with higher ploidies, albeit more likely to be missed with sparse polymorphism data (e.g. reduced-representation sequencing).

We also find that ploidy interacts with dominance to modulate the manifestation of sweep signals. In contrast with additive mutations, where there is an approximate factor-of-two effect of ploidy on fixation time, differences can be much greater for non-additive mutations, particularly when recessive (figure 1*a–c*), though we stress that this largely depends on our assumptions regarding selection and dominance. Such differences in fixation times frequently result in a reduced signal of recessive mutations in higher ploidies, whereas the effect of dominance is minimal in diploids. In other words, recessive mutations are not only more likely to be lost in higher ploidies, but they may also be more likely to go undetected when not lost (figure 2*c*; electronic supplementary material, figure S3).

This ploidy-dependent effect of dominance is partly mitigated in smaller populations. In large populations, allele frequency change due to selection can be much slower at certain allele frequencies in higher ploidies, as a consequence of the fact that genotypes with the greatest differences in relative fitness are very rare [20] (figure 1*a*–*c*). For recessive mutations, the slowdown occurs initially because the mutant homozygote bearing the full manifestation of selection is much rarer in higher ploidies (*p*^2^, *p*^4^ and *p*^8^ for dip-, tetra- and octaploids, respectively). Although greater stochasticity in allele frequency change in small populations will result in more frequent loss of these alleles, for those that survive, it may push the frequency above the critical threshold in which selection can gain traction (figure 1*b*). Once past this point, selection acts very quickly on recessive mutations, occasionally resulting in greater effects on diversity than dominant or additive mutations, in contrast with what is observed in larger populations (see points on figure 2*c* for *N* = 1000 and electronic supplementary material, figure S4). Recessive mutations will also benefit from forces which increase homozygosity (e.g. self-fertilization/inbreeding [21], double-reduction, population bottlenecks, etc.), which we have assumed are absent. Dominant mutations, on the other hand, stall at more intermediate frequencies in higher ploidies, following sharp increases in frequency early on (electronic supplementary material, figure S5). Here, drift can interfere with the weakened efficacy of selection and ultimately produce a weaker signal in genomic data.

### (b) Measures of selection

Higher ploidy routinely produced greater max values for most metrics of selection (points in figure 2*d*; electronic supplementary material, figure S6), but was inconsistent in its effects on peak area. The variance of these selection metrics also tends to decrease in higher ploidies (figure 2*e*; electronic supplementary material, figure S7), which is, again, particularly pronounced for the haplotype-based statistics. The reduced variance is not simply a consequence of sampling more chromosomes from the population (for a given number of individuals), as there is a notable reduction in variance when sampling an equivalent number of alleles (figure 2*e*). Importantly, the substantial variance in these metrics, regardless of ploidy, implies that many sweeps will go undetected in genome scans placing primacy on outlier metric values.

We also find that the signals of selection persist for a longer time in polyploids, as it takes longer to reach mutation drift equilibrium with increasing coalescent times. This was the case for the majority of the metrics that we investigated, with the exception of the iHS statistic. Interestingly, despite the increased effective recombination rate in polyploids, haplotype-based measures of selection persist longer, again reflecting polyploids' slower return to equilibrium. Recent development in phasing algorithms, necessary for calculating haplotype scores, will thus greatly advance our ability to detect selection in autopolyploids [22].

## 4. Conclusion

The numerous effects of polyploidy on fundamental aspects of evolution have multiple downstream consequences on both the evolutionary process as well as inference thereof. While increased mutational opportunity in polyploids may boost adaptation over evolutionary timescales [4], the increased sojourn time of beneficial alleles and opportunity for recombination has important consequences for shaping genomic diversity. Understanding these changes and their consequences is important, as natural polyploids are increasingly being interrogated with modern sequencing methods. In demonstrating the inherent effects of ploidy on particular population genomic measures, studies such as this can guide in the identification and interpretation of signals of selection with higher ploidy.

## Supplementary Material

Supplemental Figures

## Data accessibility

Data are available from the Dryad Digital Repository: https://doi.org/10.5061/dryad.12jm63xt1 [19]. Code for all simulation, analysis and visualizations is available at https://github.com/pmonnahan/PloidySim.
