# Supplementary material for: The effect of autopolyploidy on population genetic signals of hard sweeps
Source: Biol Lett. 2020 Feb 26;16(2):20190796. doi: 10.1098/rsbl.2019.0796 (PMC7058959; doi:10.1098/rsbl.2019.0796)

The effect of autopolyploidy on population genetic signals of hard sweeps

Patrick Monnahan^1^ and Yaniv Brandvain^1^

^1^ Microbial and Plant Genetics Institute, University of Minnesota, St. Paul, MN 55108

Corresponding author: Patrick Monnahan, [pmonnaha@umn.edu](mailto:pmonnaha@umn.edu)

Word Count: 2499

Keywords: polyploidy, hitchhiking, linked selection, hard sweep

Running title: Autopolyploidy and hard selective sweeps

**Supplemental Figures:**

Supplemental Figure 1. Coalescent simulation framework. The coalescent time unit equals *k * N*, and the population mutation and recombination rates equal *θ=2NkμL* and ρ*=2NkrL*, respectively, where *L* is the length of sequence to simulate (*L =* 1 Mb and *μ = r* for all simulations). We set *μ = r* = 1x10^-8^. We initially varied these two parameters independently, but discovered that varying *μ* simply set the baseline level of diversity and did not qualitatively alter any of the findings. Although we investigated a range of values for fuseTime, it was set to 1 generation (immediately preceding the start of selection) for all results in paper because we found nothing of interest to report for alternative values. sampGen is the same as *G_s_* in the manuscript.


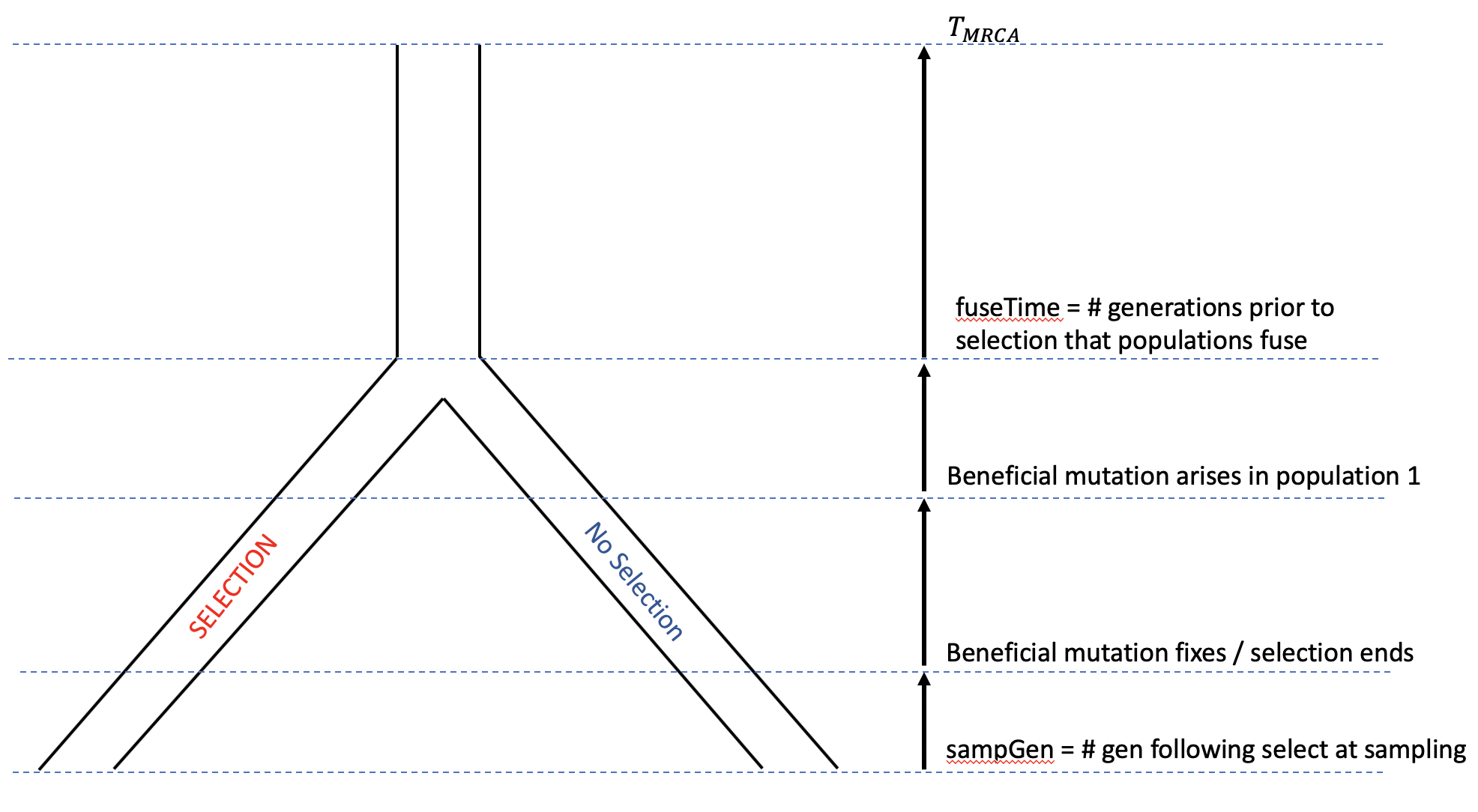


We implement the above scenario with the following command to mssel:

~/.mssel <nsam> <nreps> <nanc> <nder> <trajectory_file> <sel_spot> -r <rho> <length> -t <theta> -I 2 0 <nder> <nanc> 0 -ej <fuseTime> 2 1

Where brackets denote specification of a parameter and parameters that were varied are,

nsam = Number of sample alleles/haplotypes; equals *n * k*, where *n* equals the number of sampled individuals and *k* equals the ploidy level.

nreps = Number of replicates; though this was set to 1 for all simulations because we only want one simulation per trajectory file, and we achieve replication by using multiple independent-generated trajectory files.

nanc = Number of haplotypes with the ancestral (non-selected) allele; equals (*n* * *k*) / 2

nder = Number of haplotypes with the derived (selected) allele; also equals (*n* * *k*) / 2

trajectory_file = path to text file containing the allele frequency trajectory simulated via scheme in Methods. Also, see PloidyHitch.R at <https://github.com/pmonnahan/PloidySim>

for implementation

sel_spot = position of the selected mutation

rho = population recombination rate; ρ*=2NkrL*

length = number of sites

theta = population diversity rate; *θ=2NkμL*

The parameters following the flag, -I, specify the demographic scenario with the form “npop n1anc n1der n2anc n2der”, where,

npop = number of populations (always 2 in our case)

n1anc = number of ancestral alleles sampled from population 1. We set this to 0 because selection has fixed the derived allele in population 1.

n1der = number of derived alleles sampled from population 1. Since the derived allele is fixed in this population, we want to sample entirely derived alleles from this population.

n2anc = number of ancestral alleles sampled from population 2. Since the derived allele arises in population 1 following the split of the ancestral population, the derived allele is not present in population 2, and thus we specify only sampling haplotypes with the ancestral allele in population 2

n2der = number of derived alleles sampled from population 2. Always set to 0.

The parameters following the flag, -ej, specify the coalescent time (fuseTime in the figure and command; in units *k * N*) at which the two populations fused. The “2 1” argument specifies that population 2 fuses into population 1, although this choice is arbitrary and does not affect the results in any way.

Supplemental Figure 2. Line plots for individual replicates when s = 0.1 (top) and 0.01 (bottom). We provide these figures to illustrate the degree of variation across replicates for Figure 2A. Note that variation in red and green lines is partially obscured by the top blue layer, which show a similar degree of variation as in blue.


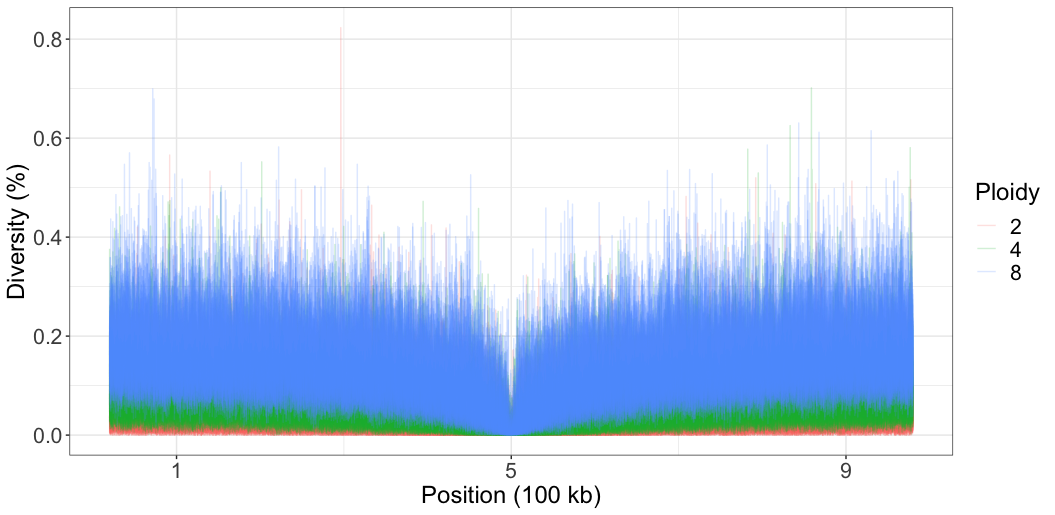


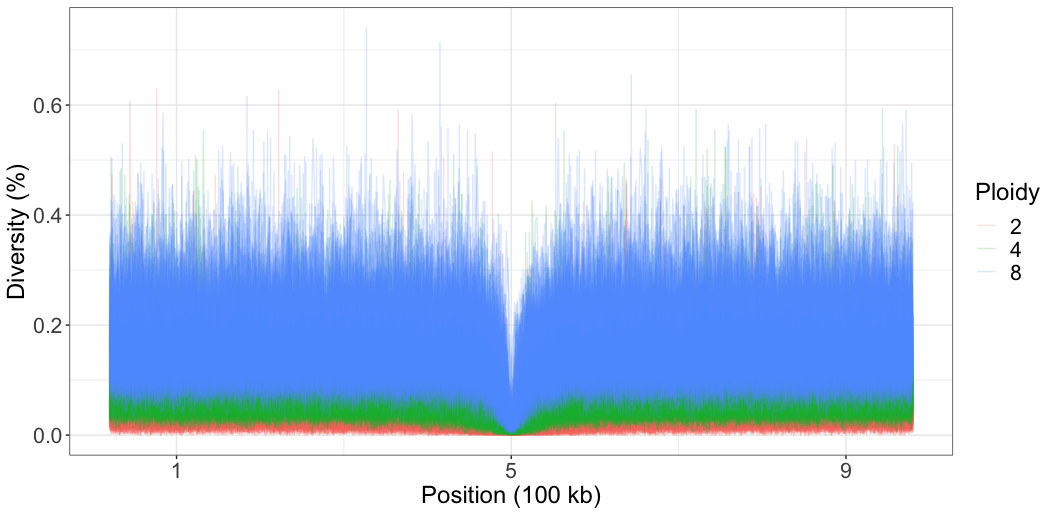


Supplemental Figure 3. Top: Bottom; Effect of dominance on various metrics of selection when N=10,000 for different ploidy levels. Points (X’s) denote scaled-median of peak area for N=1,000. Area is calculated as the Magnitude * Breadth / 2 as described in Methods. The ‘Diversity’ panel is the same as presented in Figure 2C.

**
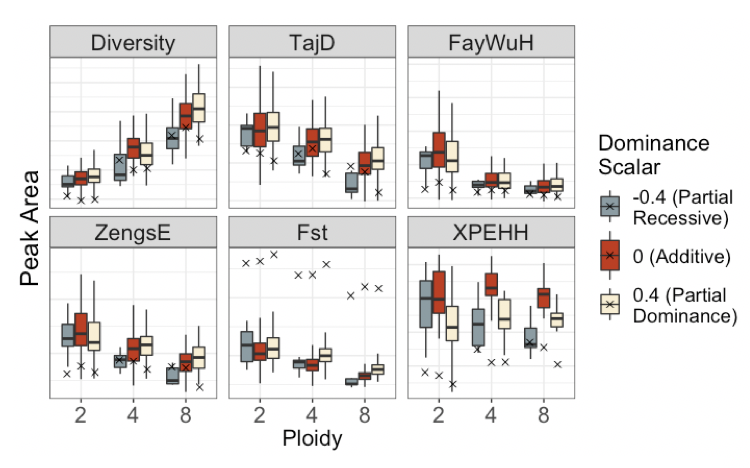
**

Supplemental Figure 4. This figure presents the full data for N=1,000 to provide additional detail to Figure 2C, where the left panel is simply represented by a point for the median. Also, the left panel lacks the multiplication by 10 as was done in 2C to bring N=1000 and N=10000 on the same scale.


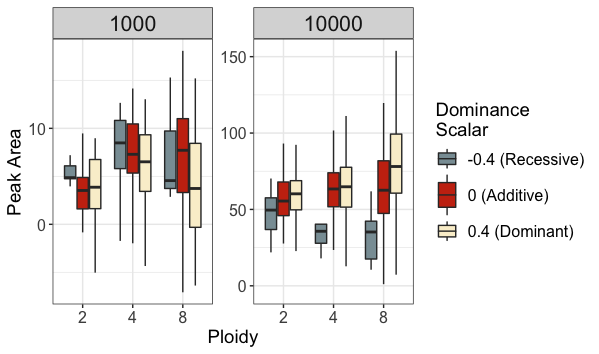


Supplemental Figure 5. Allele frequency trajectories for different ploidy levels for dominant and additive mutations. s = 0.1, starting frequency = 0.05. Compared to additive mutations, dominant mutations generally take longer to fix, regardless of ploidy, but the difference in fixation time becomes more pronounced at higher ploidy.


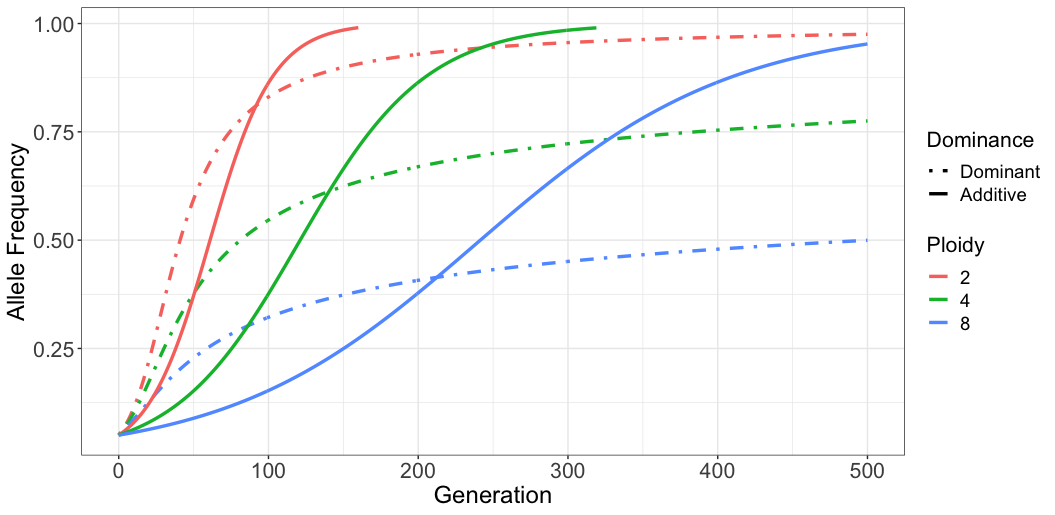


Supplemental Figure 6. Effect of sampling generation (see Supp. Figure 1) on selection metrics for different ploidy levels. Top: H = -0.4 (partially recessive) and Bottom: H = 0.4 (partially dominant). Points denote median across replicates of maximum observed values, and boxplots are integrated area under the curve. The bottom figure is based on 100 replicates, while the top figure is based on 30 replicates due to high computational costs of simulating recessive mutations.


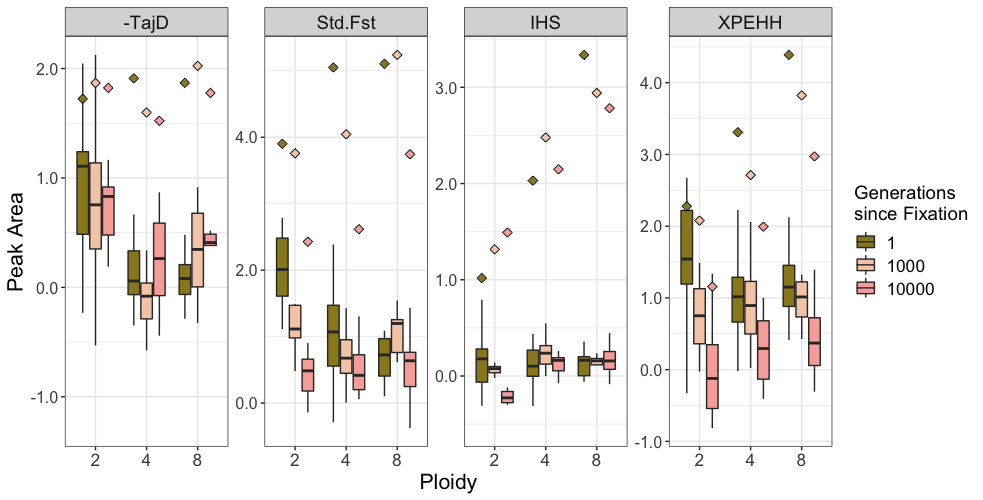


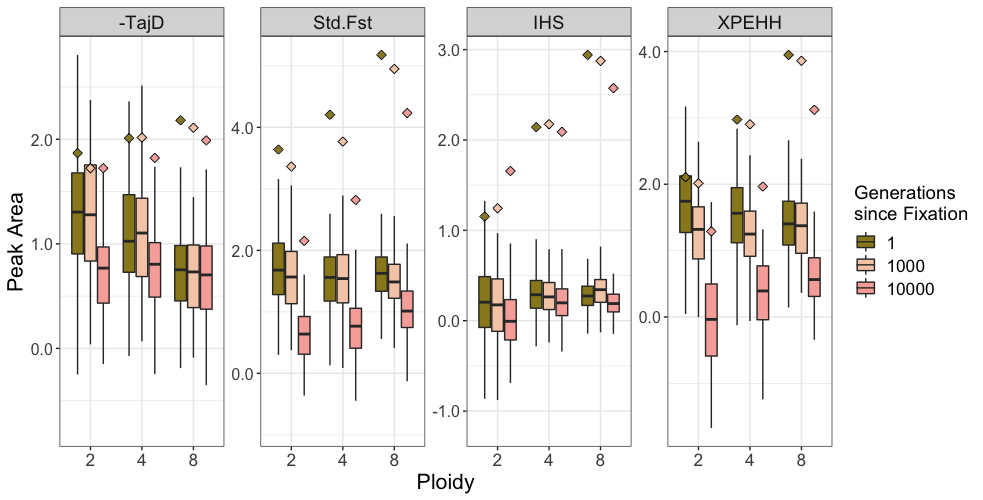


Supplemental Figure 7. Effect of ploidy and sampling regime on the amount of variation (as measured by the coefficient of variation; CV) for different ploidy levels. Top: H = -0.4 (partially recessive) and Bottom: H = 0.4 (partially dominant). Partially recessive mutations are much more computationally expensive to simulate, so results are based on fewer replicates (40 reps; error bars are from resampling 5 reps, 1000 times) compared to dominant mutations (1000 reps; error bars from resampling 100 reps, 1000 times).


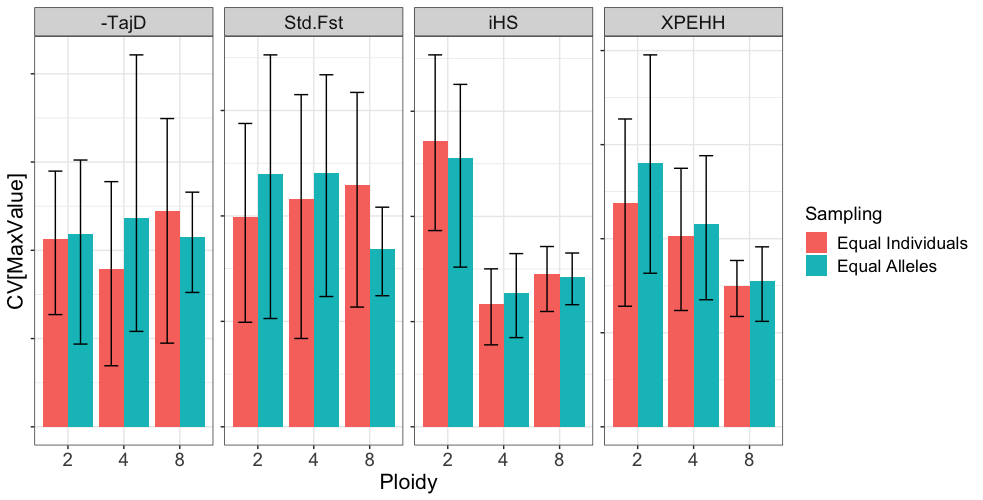


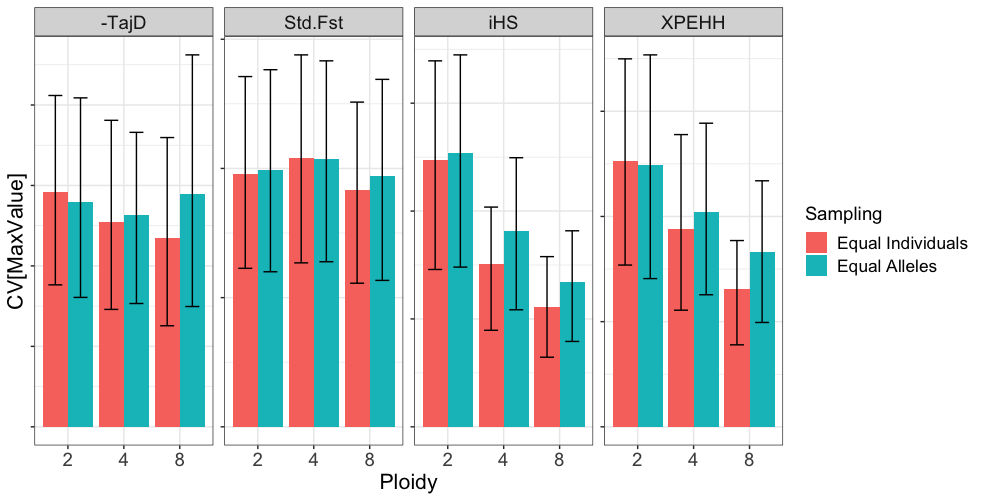

Supplement: Supplemental Figures [file rsbl20190796supp1.docx]
